# One Index Does Not Predict All—Hematological Derived Indices Have Different Predictive Value for ICU Mortality in Critically Ill Patients with Non-Infectious Versus Infectious Acute Exacerbation of COPD

**DOI:** 10.3390/medicina62040728

**Published:** 2026-04-10

**Authors:** Emanuel Moisa, Silvius Ioan Negoita, Claudia Mihail, Liviu Ioan Serban, Alexandru Tudor Steriade, Cristian Cobilinschi, Madalina Dutu, Georgeana Tuculeanu, Dan Corneci

**Affiliations:** 1 Department of Anesthesiology and Critical Care, Faculty of Medicine, ‘Carol Davila’ University of Medicine and Pharmacy, 020021 Bucharest, Romania; emanuel.moisa@umfcd.ro (E.M.); cristian.cobilinschi@umfcd.ro (C.C.); madalina.dutu@umfcd.ro (M.D.); georgeana.tuculeanu@drd.umfcd.ro (G.T.); dan.corneci@umfcd.ro (D.C.); 2 Department of Anesthesiology and Critical Care, Elias University Emergency Hospital, 011461 Bucharest, Romania; claudia.mihail@drd.umfcd.ro (C.M.); liviuioanserban@gmail.com (L.I.S.); 3 Department of Cardio-Thoracic Pathology, Faculty of Medicine, ‘Carol Davila’ University of Medicine and Pharmacy, 020021 Bucharest, Romania; alexandru.steriade@umfcd.ro; 4 Department of Pneumology and Acute Respiratory Care, Elias University Emergency Hospital, 011461 Bucharest, Romania; 5 Department of Anesthesiology and Critical Care, Clinical Emergency Hospital, 010024 Bucharest, Romania; 6 Department of Anesthesiology and Critical Care, Dr. Carol Davila Central Military Emergency University Hospital, 010825 Bucharest, Romania

**Keywords:** SIRI, MLR, NLR, SII, COPD, ICU, acute exacerbation, AECOPD, mechanical ventilation, mortality

## Abstract

*Background and Objectives*: Acute exacerbation of COPD (AECOPD) poses a major burden on healthcare systems, with critically ill AECOPD patients having increased morbidity and mortality. Since adverse outcomes are due both to respiratory failure and the systemic inflammatory response, prognostic markers accounting for these patterns are needed. Our aim was to investigate the predictive power of derived hematological indices for intensive care unit (ICU) mortality in patients with non-infectious versus infectious AECOPD. *Materials and Methods*: This is a retrospective, observational, monocentric cohort study on 88 AECOPD patients admitted to the ICU between 2018 and 2023. Descriptive statistics were performed for the entire cohort, and for predefined subgroups (non-infectious, infectious and bacterial AECOPD). Receiver Operating Characteristics (ROC) analysis was performed to test the predictive power of the studied indices. Cut-off values were identified using the Youden index. Kaplan–Meier analysis was conducted to test the association with ICU mortality. *Results*: Overall ICU mortality was 44%. For the whole cohort, neutrophil-to-lymphocyte ratio (NLR), neutrophil-to-platelets ratio (NPR) and systemic inflammation response index (SIRI) showed moderate predictive power for ICU mortality (areas under the curve (AUCs) of 0.71–0.73). Non-infectious and infectious subgroups were comparable in terms of size, demographics, comorbidities and baseline COPD characteristics (*p* > 0.05). Mortality was significantly higher in infectious AECOPD (64.6% versus 20%, *p* < 0.001). For non-infectious AECOPD, monocyte-to-lymphocyte ratio (MLR) and SIRI had very good predictive power (AUCs between 0.82 and 0.855), while NPR and systemic inflammation index (SII) showed moderate AUC values (between 0.7 and 0.79). In infectious AECOPD, only NPR retained fair predictive power (AUC 0.691), which improved in bacterial AECOPD (AUC 0.781). *Conclusions*: Derived hematological indices have different predictive values for ICU mortality. MLR and SIRI exhibited very good predictive power in non-infectious AECOPD, while NPR was the best discriminator in bacterial AECOPD. These stress the importance of individualized prognostication in AECOPD.

## 1. Introduction

An acute exacerbation of chronic obstructive pulmonary disease (AECOPD) is defined as an event characterized by increased dyspnea and/or cough and sputum that worsens over <14 days [1]. The natural course of the disease is characterized by a progressive evolution, with periods of clinical stability alternating with recurrent acute exacerbations. Notably, AECOPD is a leading cause of morbidity and mortality and accounts for a substantial proportion of the global burden of COPD [2]. In-hospital mortality rates range between 11% and 33% [3,4], being significantly higher among patients admitted to intensive care units (ICU) [5]. The etiology of acute exacerbations in patients requiring ICU admission is frequently infectious (60–80%), while non-infectious AECOPD accounts for 20–40% of cases [5].

Admission to the ICU among hospitalized AECOPD was inconsistently reported across studies, with rates varying between 5% and 19% [5,6]. This variation is probably explained by limitations related to heterogenous selection criteria, COPD misdiagnosis and healthcare systems shortcomings [5]. Given the significant burden imposed by AECOPD on healthcare systems, defining reliable prognostic factors for adverse outcomes is essential, yet remains a key clinical challenge. Evidence regarding the epidemiological profile and independent predictors of mortality based on etiology (infectious versus non-infectious) or inflammatory patterns remains limited [7].

For critically ill AECOPD patients, there are several studies reporting that systemic inflammatory response is a major contributing factor to mortality in this population [8,9]. This is in line with the hallmark of COPD pathogenesis—dynamic interactions between innate and adaptive pathways, leading to a sustained local inflammation as well as a systemic inflammatory response [10,11]. In most severe cases, these pathways contribute not only to the severity of respiratory failure [12,13], but also to systemic physiological disturbances and multi-organ failure [10,14]. Given these considerations, the clinical outcome is ultimately dictated not only by pre-existing conditions or the need for mechanical ventilation, but also by the severity of acute organ dysfunctions at admission. Therefore, identifying reliable early prognostic markers could facilitate prompter interventions and a personalized therapeutic approach [10].

Emerging evidence indicates that derived hematological indices like the neutrophil-to-lymphocyte ratio (NLR), derived NLR (dNLR), neutrophil-to-platelet ratio (NPR), platelet-to-lymphocyte ratio (PLR), monocyte-to-lymphocyte ratio (MLR), systemic inflammation index (SII) and systemic inflammation response index (SIRI) are associated with higher mortality rates, increased disease severity and new organ dysfunctions in various pathologic states [15,16,17]. Although hematologic parameters are inexpensive and routinely measured in patients requiring ICU admission, the prognostic value of these markers remains under-studied and inconsistent, and no clear cut-off values have been established. A few studies investigated these indices in AECOPD as diagnostic or prognostic markers [18,19,20]. Considering the intricate inflammatory response in critically ill AECOPD, the prognostic value of indices is worth studying according to the etiology of exacerbation—infectious versus non-infectious.

Therefore, the aim of this study was to evaluate the predictive value for ICU mortality of different derived hematological indices measured at ICU admission, in critically ill patients with AECOPD of infectious and non-infectious etiology.

## 2. Materials and Methods

### 2.1. Study Design and Population

This is a retrospective, observational, cohort study from a single center (Elias Emergency University Hospital of Bucharest, Romania) and included critically ill patients with AECOPD admitted to the ICU between January 2018 and June 2023. This study was approved by the Local Ethics Committee (Reference number: 10108-1 from 1 August 2023). Inclusion criteria were: age ≥ 18 years old and AECOPD requiring ICU admission. Acute exacerbation of AECOPD was diagnosed according to the available practice guidelines at the respective time [1,21,22] and always included an assessment performed by at least a pneumologist and an intensivist. Infectious and non-infectious etiology of AECOPD was determined based on a combination of: (1) clinical examination, (2) radiological and/or imagistic findings (chest X-ray or computed tomography, cardiac echography), (3) history of exposure to trigger factors, (4) adherence to treatment, and (5) microbiological and laboratory tests. Moreover, to correctly classify patients into one group the following were performed: (1) review of patient’s record diagnoses (the diagnosis of infectious or non-infectious AECOPD was specified in all records), (2) review the electronically written diagnoses or diagnostic codes supporting AECOPD type (e.g., bacterial or viral pneumonia, cardiovascular events, environmental exposure, etc.), (3) review of all microbiological tests performed (urinary antigens for *Streptococcus pneumoniae* and *Legionella pneumophila*, sputum, tracheal aspirate or broncho-alveolar lavage cultures, polymerase chain reaction tests for viruses or bacteria). A diagnosis of bacterial AECOPD was made in patients with positive cultures or tests for a respiratory bacterial infection, together with clinical, radiological and laboratory exams. The same approach was used in viral AECOPD. Exclusion criteria were: (1) patients admitted in another department than ICU (high dependency units were not included), (2) end-stage oncological or organ disease (heart, kidney, liver), (3) patients with ongoing radio-, chemo- or immune therapy, (4) severe hematological and (5) autoimmune diseases, (6) patients transferred to another hospital and (7) ICU readmission.

### 2.2. Study Outcomes

Primary outcome: Assessment of the predictive power of hematological parameters and derived indices for ICU mortality in patients with non-infectious versus infectious AECOPD.

Secondary outcomes:

- Assessment of the predictive power of hematological parameters and derived indices for ICU mortality in patients with bacterial AECOPD.
- Assessment of the predictive power of non-hematological predictors for ICU mortality.

### 2.3. Data Collection

Data collected in this study included: demographic (age, gender), COPD and non-COPD-related comorbidities, Charlson Comorbidity Index (CCI), COPD characteristics (GOLD stage, number of exacerbations per year, at-home oxygen therapy or non-invasive mechanical ventilation requirement), smoking status, professional exposure, and clinical and laboratory data at ICU admission. Clinical data was collected at ICU admission and included: type of AECOPD, type of respiratory support (invasive or non-invasive mechanical ventilation), Glasgow Coma Scale (GCS) and severity and risk scores (SOFA and APACHE II). Clinical data at ICU admission or during ICU LOS included the frequency of progression to invasive mechanical ventilation, need for vasopressor agents, use of hypnotics, opioids and neuromuscular blocking agents (NMBA), development of acute kidney injury (AKI), use of bronchodilators and the need for tracheostomy. These variables were binary coded (yes or no) and no data about drug doses were collected. Moreover, ICU length of stay (LOS) and mortality rate were recorded. Laboratory data included: parameters from hemogram (absolute count of leukocytes, neutrophils, monocytes, eosinophils, basophils, platelets and erythroblasts, hemoglobin, haematocrit, mean corpuscular volume (MCV), mean corpuscular hemoglobin (MCH) and mean corpuscular hemoglobin concentration (MCHC), red blood cell distribution width (RDW)) and their derived indices (defined later), sodium, creatinine, urea, bilirubin, arterial partial pressure of CO_2_ (P_a_CO_2_), P_a_O_2_/FiO_2_ ratio, blood lactate, C-reactive protein (CRP) and procalcitonin. For C-reactive protein and procalcitonin, only 51 (58%) and 77 (87.5%) measurements, respectively, were available at ICU admission. For all the other parameters, no missing values were registered.

### 2.4. Definitions

The following hematological indices were derived from the complete blood count analysis: (1) derived neutrophil-to-lymphocyte ratio (dNLR, (absolute neutrophil count/absolute leukocyte count—absolute neutrophil count)), (2) neutrophil-to-lymphocyte ratio (NLR), neutrophil-to-platelets ratio (NPR), systemic inflammation index (SII, (absolute neutrophil count × absolute platelet count)/absolute lymphocyte count), monocyte-to-lymphocyte ratio (MLR), systemic inflammation response index (SIRI, neutrophils x monocytes/lymphocytes) and platelets-to-lymphocytes ratio (PLR).

### 2.5. Statistical Analysis

For this analysis, the IBM Statistical Package for Social Sciences (SPSS) for Windows^®^, version 30.0 (IBM Corp., Armonk, NY, USA) and MedCalc Software^®^, version 20.106 (Ostend, Belgium) were used. Following the Shapiro–Wilk test, continuous data were reported as mean ± standard deviation (SD) or median and interquartile range [IQR; Q1, Q3]. Comparison of continuous data with parametric or nonparametric distribution between two independent groups was performed using the independent *t*-test or the Mann–Whitney test. Categorical data were expressed as absolute and relative frequencies and comparisons were conducted using Chi-square or Fisher’s exact tests accordingly. Receiver Operating Characteristics (ROC) analysis was performed to test the predictive power of the parameters studied. Area under the curve (AUC or AUROC) was reported together with the corresponding 95% confidence intervals (CI). Cut-off values were identified using the Youden index. Sensitivity, specificity, positive and negative likelihood ratios (LR) were calculated for every cut-off value. Comparisons between ROC curves were performed with the DeLong test. Kaplan–Meier analysis was conducted to test the association with ICU mortality during ICU LOS. Hazard ratios (HR) with 95% CIs and the chi-square value of the log-rank test were reported for every model. Multivariable binary logistic regression was used to test the independent predictive power of the studied indices. Univariable analyses were performed for all possible predictors. Multivariable models included the studied indices and the possible confounding factors identified as significant in univariable analysis or based on clinical reasoning. Multicollinearity diagnostics were performed and factors with a tolerance of <0.2 and/or a variation inflation factor of >5 were not included in the models to avoid overfitting. The method for variable entry in the models was Enter. The Hosmer–Lemeshow test was performed to assess model calibration. Nagelkerke R square, overall prediction, beta value, standard error, Wald, odds ratio and 95% CI were reported for all models. An alpha level of less than 0.05 was considered significant and all tests were two-tailed.

All the results were reported in accordance with the STROBE (Strengthening the Reporting of Observational Studies in Epidemiology) checklist [23] (Supplementary Materials S1).

## 3. Results

For this analysis, 88 critically ill AECOPD patients were included, with 59 (67%) males and a mean age of 66.8 (±10.67) years. Out of these 88 patients, 40 (45.5%) and 48 (54.5%) had non-infectious and infectious AECOPD, respectively. In the infectious AECOPD group, the etiology was bacterial in 40 (83.3%) cases and viral in 8 (16.6%) cases.

Further results will be presented with respect to the type of AECOPD—non-infectious versus infectious (Table 1). There was no significant difference between groups for gender, age, associated diseases or median value of CCI score (*p* > 0.05). Moreover, the two groups did not differ in terms of COPD characteristics such as smoking status, professional exposure, GOLD stage, number of exacerbations or home respiratory support. At ICU admission, patients with infectious AECOPD had higher median SOFA (7 [4–9.75] versus 5 [3–7.5], *p* = 0.034), APACHE II (20.5 [16.25–31.25] versus 18 [14–22], *p* = 0.046), procalcitonin (1.27 [0.28–4.5] versus 0.18 [0.09–0.5], *p* < 0.001) and CRP (86 [46.5–241.5] versus 16.1 [8–28.5], *p* < 0.001). No difference in IMV versus NIV requirement at ICU admission was observed between groups, but patients with infectious AECOPD required IMV more frequently during ICU LOS (23 (72%) versus 13 (46.4%), *p* = 0.045) and had a longer median duration of IMV (111 [33–213] versus 15.5 [0–183], *p* = 0.019), higher need for NMBA (21 (43.8%) versus 7 (17.5%), *p* = 0.008) and vasopressor agents (36 (75%) versus 13 (32.5%)). Four patients required two vasopressor drugs and two required inotropic agents (dobutamine).

Regarding the hematological parameters and derived indices, patients with infectious AECOPD had significantly higher median values for leukocytes (*p* = 0.002), neutrophils (*p* < 0.001), dNLR (*p* < 0.001), NLR (*p* < 0.001), NPR (*p* = 0.023), SII (*p* < 0.001), PLR (*p* < 0.001), MLR (*p* = 0.01) and SIRI (*p* < 0.001) and lower median values for lymphocytes (*p* < 0.001) and eosinophils (*p* = 0.041). There was no difference between groups for all the other laboratory measurements (*p* > 0.05).

**Figure 1:**
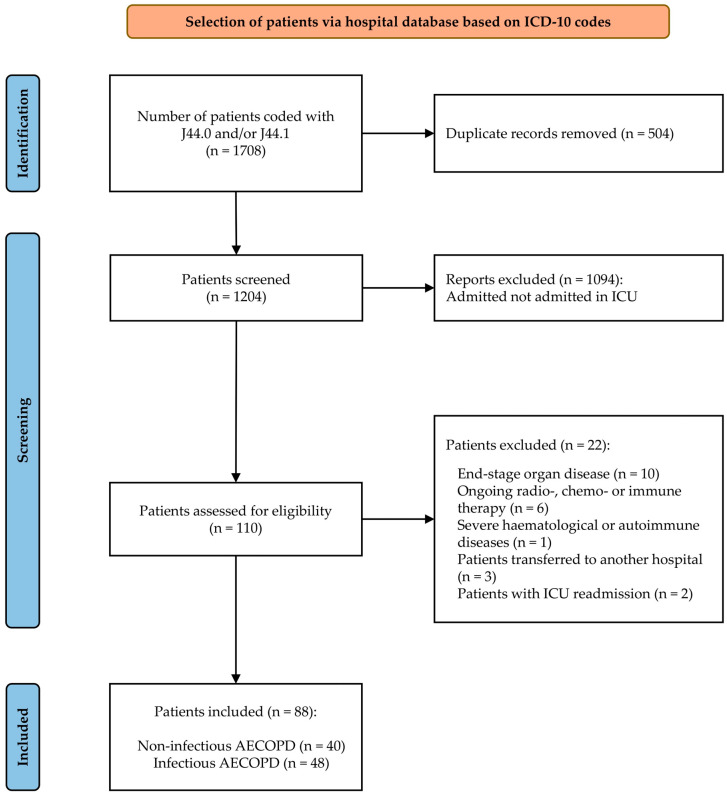
Flowchart of the study population.

Lastly, no difference for ICU LOS or hospital LOS was observed, but patients with infectious AECOPD had a significantly higher mortality rate (31 (64.6%) versus 8 (20%), *p* < 0.001) (Figure 1).

### 3.1. Discriminative Power Analysis (ROC) of the Hematological and Non-Hematological Parameters for ICU Mortality

The predictive power of hematological parameters and derived indices was tested for the whole sample and depending on the type of AECOPD: non-infectious, infectious and bacterial subtype. The full analysis is reported in Table 2.

#### 3.1.1. ROC Analysis for All AECOPD Patients

The ROC analysis revealed that regardless of AECOPD type, neutrophils, NLR, NPR and SIRI have modest predictive power for ICU mortality with AUCs between 0.71 and 0.73, while leukocytes, dNLR, SII, MLR, haematocrit and MCHC had fair discriminative ability with AUCs between 0.61 and 0.696 for the same studied outcome (Table 2). NLR had the best sensitivity (87.2%) for a cut-off of >10.12, while dNLR had the best specificity (87.8%) for a cut-off of >10.53. All cut-off values, sensitivity, specificity, as well as the positive and negative LR are reported in Table 3.

Of the non-hematological parameters included in the analysis, SOFA and APACHEII scores, urea and lactate also had significant predictive power for ICU mortality, with SOFA having the highest AUC of all parameters (Table 2). Pairwise comparisons of all ROC curves found no significant difference (*p* > 0.05).

#### 3.1.2. ROC Analysis in Non-Infectious AECOPD Patients

In patients with non-infectious AECOPD the predictive power was moderate for leukocytes, neutrophils, monocytes, NPR and SII (AUC values between 0.7 and 0.79) and very good for MLR and SIRI, respectively (AUC values between 0.82 and 0.855) (Table 2). For the identified cut-off values, leukocytes and NPR had the best sensitivity, of 100%, while monocytes and SIRI had the best specificity of 87.5%. The complete analysis is available in Table 3. Apart from GCS score, no other non-hematological parameter had significant predictive power (Table 2). The AUROC value for SIRI was significantly higher than the AUROC of SII (*p* = 0.29). For all the other comparisons, no difference was found.

#### 3.1.3. ROC Analysis in Infectious AECOPD Patients

In patients with infectious AECOPD, platelets and MCHC had moderate predictive power, while for NPR, it was only fair (Table 2). For a cut-off of >31.5, MCHC had the best sensitivity and specificity of 64.5% and 88.2%, respectively (Table 3). SOFA and APACHE II scores, as well as lactate and P_a_CO_2_ had significant predictive value, with SOFA having the highest AUC (Table 2). Pairwise comparisons of all ROC curves found no significant difference (*p* > 0.05).

#### 3.1.4. ROC Analysis in Bacterial AECOPD Patients

In bacterial AECOPD, only NPR had moderate predictive value, while leukocytes, neutrophils, platelets and MCHC had fair discriminative ability (Table 2). Leukocytes had the best sensitivity (88%), while MCHC had the best specificity (88.7%) (Table 3). As with infectious AECOPD, SOFA had the best predictive power. APACHE II and lactate were also significantly associated with ICU mortality (Table 2). Pairwise comparisons of all ROC curves found no significant difference (*p* > 0.05).

### 3.2. Kaplan–Meier Analysis for ICU Mortality Prediction During LOS

Kaplan–Meier analysis was conducted for all the hematological and derived indices found to have a significant AUC (Table 4).

**Figure 2:**
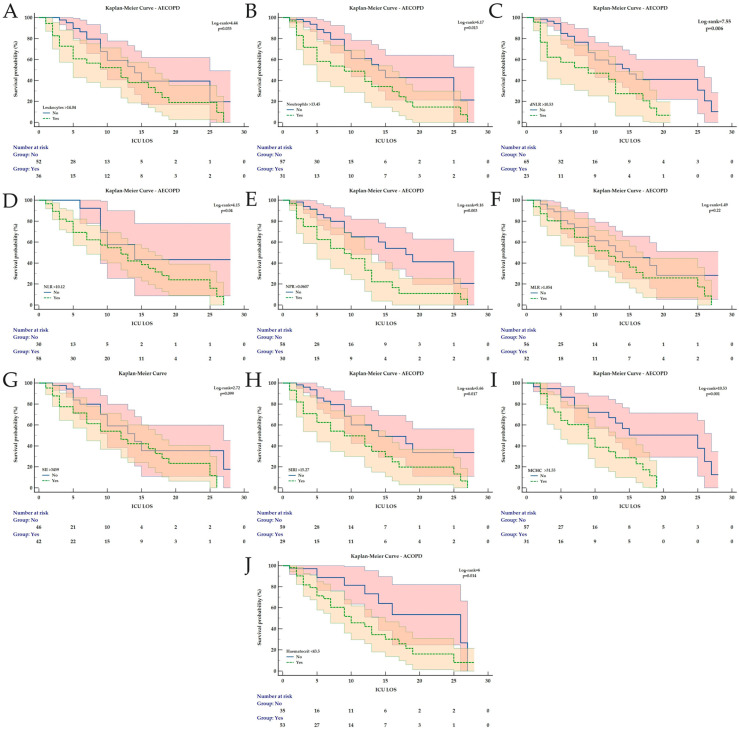
Kaplan–Meier Curves for ICU mortality during ICU LOS in all AECOPD patients depending on the identified cut-off values for (**A**) Leukocytes, (**B**) Neutrophils, (**C**) dNLR, (**D**) NLR, (**E**) NPR, (**F**) MLR, (**G**) SII, (**H**) SIRI, (**I**) MCHC and (**J**) Haematocrit. All curves are expressed with the corresponding 95% confidence intervals. The Chi-square value of the Log-rank test and *p* value are reported. Abbreviations: AECOPD = Acute Exacerbation of Chronic Obstructive Pulmonary Disease; dNLR = derived Neutrophil-to-Lymphocyte Ratio; ICU = Intensive Care Unit; LOS = Length of Stay; MCHC = Mean Corpuscular Hemoglobin Concentration; MLR = Monocyte-to-Lymphocyte ratio; NLR = Neutrophil-to-Lymphocyte Ratio; NPR = Neutrophil-to-Platelet Ratio; SII = Systemic Inflammation Index; SIRI = Systemic Inflammation Response Index.

**Figure 3:**
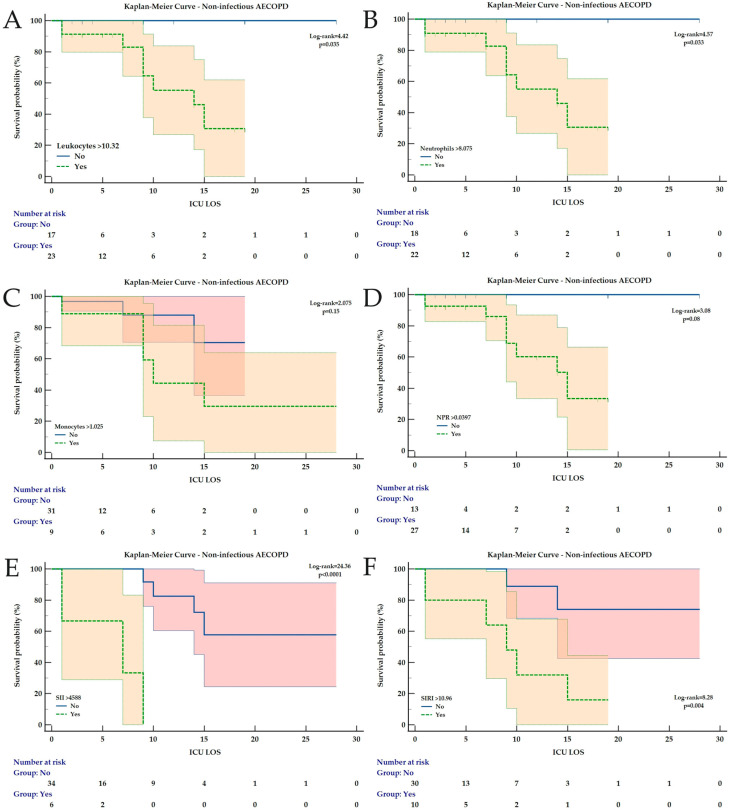
Kaplan–Meier Curves for ICU mortality during ICU LOS in non-infectious AECOPD for (**A**) Leukocytes, (**B**) Neutrophils, (**C**) Monocytes, (**D**) NPR, (**E**) SII and (**F**) SIRI. All curves are expressed with the corresponding 95% confidence intervals. The Chi-square value of the Log-rank test and *p* value are reported. In images (**A**,**B**,**D**) for patients from the “No” subgroup, all cases were censored as the studied event (ICU mortality) did not occur in any patient. Abbreviations: AECOPD = Acute Exacerbation of Chronic Obstructive Pulmonary Disease; ICU = Intensive Care Unit; LOS = Length of Stay; NPR = Neutrophil-to-Platelet Ratio; SII = Systemic Inflammation Index; SIRI = Systemic Inflammation Response Index.

**Figure 4:**
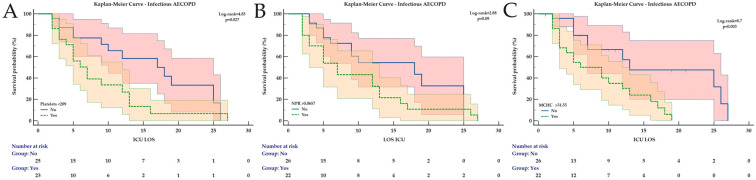
Kaplan–Meier Curves for ICU mortality during ICU LOS in infectious AECOPD for (**A**) Platelets, (**B**) NPR and (**C**) MCHC. All curves are expressed with the corresponding 95% confidence intervals. The Chi-square value of the Log-rank test and *p* value are reported. Abbreviations: AECOPD = Acute Exacerbation of Chronic Obstructive Pulmonary Disease; ICU = Intensive Care Unit; LOS = Length of Stay; MCHC = Mean Corpuscular Hemoglobin Concentration; NPR = Neutrophil-to-Platelet Ratio.

**Figure 5:**
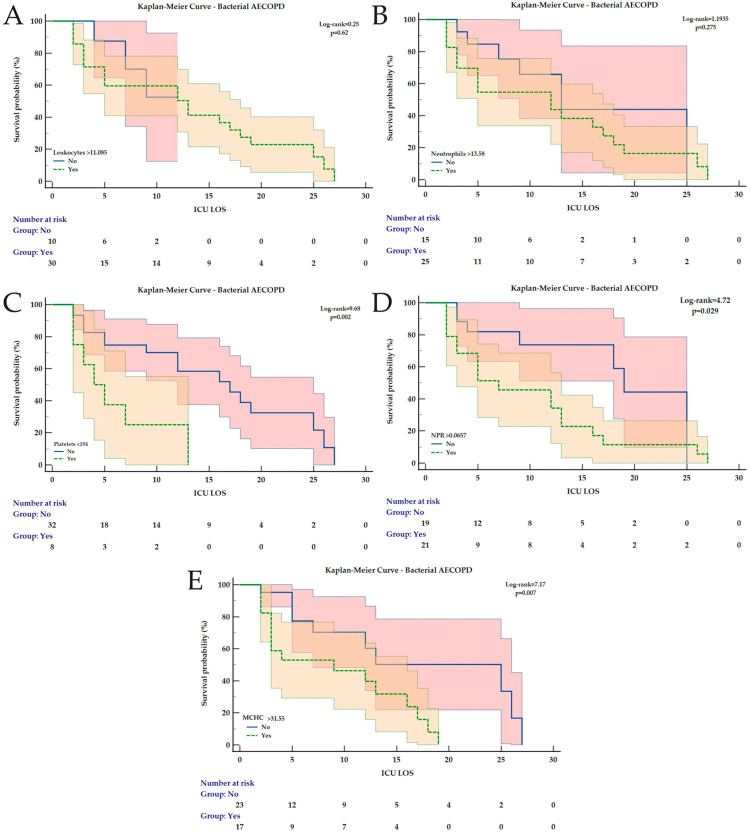
Kaplan–Meier Curves for ICU mortality during ICU LOS in bacterial AECOPD for (**A**) Leukocytes, (**B**) Neutrophils, (**C**) Platelets, (**D**) NPR and (**E**) MCHC. All curves are expressed with the corresponding 95% confidence intervals. The Chi-square value of the Log-rank test and *p* value are reported. Abbreviations: AECOPD = Acute Exacerbation of Chronic Obstructive Pulmonary Disease; ICU = Intensive Care Unit; LOS = Length of Stay; MCHC = Mean Corpuscular Hemoglobin Concentration; NPR = Neutrophil-to-Platelet Ratio.

For all AECOPD patients, at the identified cut-off values, all parameters remained significantly associated with ICU mortality during LOS, except for MLR and SII (Figure 2). For non-infectious AECOPD, only MLR was not significantly associated with ICU mortality (Figure 3). In patients with infectious AECOPD, NPR was not associated with ICU mortality (Figure 4), while the same observation was made for leukocytes and neutrophils in bacterial AECOPD patients (Figure 5). The full analysis, including the HRs with the corresponding 95% CIs, is available in Table 4 and Figure 2, Figure 3, Figure 4 and Figure 5.

### 3.3. Multivariable Analysis for ICU Mortality Prediction

Multivariable binary logistic regressions were performed for all studied indices. In all AECOPD patients, only neutrophils, NPR, SIRI and MCHC retained their independent predictive value. In these patients, hematological indices were adjusted for gender, Charlson Comorbidity Index, GOLD stage, type of exacerbation and SOFA score.

For the non-infectious AECOPD subgroup, hematological indices were adjusted for gender, Charlson Comorbidity Index and Glasgow Coma Scale. In this subgroup, only SIRI, SII and monocytes retained their independent predictive value, while for leukocytes, neutrophils, NPR and MLR, no models were computed as the studied event appeared with a frequency of 100% in one of the subgroups.

For infectious AECOPD, the following confounders were introduced in the models: gender, age, history of left ventricular heart failure and SOFA score. For the model including the predictive value of platelets, a modified SOFA was introduced. We excluded the points given for thrombocytopaenia in the SOFA score to avoid multicollinearity. Finally, NPR and MCHC were independently associated with ICU mortality.

In patients with bacterial AECOPD, hematological indices were adjusted for gender, Charlson Comorbidity Index and SOFA score. Leukocytes, neutrophils, NPR and MCHC were independently associated with the studied outcome. The full models for all indices are reported in Supplementary Material S2.

## 4. Discussion

While AECOPD has been widely studied, the critically ill population has been insufficiently assessed. To the best of our knowledge, this study provides the first comparative analysis on infectious versus non-infectious AECOPD patients admitted to the ICU in a Romanian cohort. Derived hematological indices—NLR, NPR and SIRI—exhibited moderate discriminative performance as prognostic factors for ICU mortality among all AECOPD patients, as reflected by their AUROCs. Notably, subgroup analysis revealed improved AUROC values and very good discriminative power for MLR and SIRI in the non-infectious cohort, with SIRI being the best performing index in this subgroup (AUROC 0.855, 95% CI 0.71–1). Conversely, in AECOPD of bacterial infectious etiology, only NPR retained good discriminatory value, with an AUROC of 0.781, 95% CI 0.64–0.93.

The incidence of ICU admission in our study was 9.14%, which is consistent with previously reported data [5]. Notably, the final cohort comprised ICU patients with a high severity of illness as reflected by the need for mechanical ventilation in all subjects (with 60% of them IMV during LOS) and by organ dysfunction scores (median SOFA and APACHE II scores of 6 and 19, respectively). Considering that more severe AECOPD patients show distinct patterns of the studied hematological indices [24], the aforementioned aspects enhance the generalizability of our results to high-severity ICU populations, rather than mixed cohorts including patients ventilated in high-dependency units.

As one of the few observational studies investigating prognostic markers according to the etiology of AECOPD, our retrospective cohort demonstrated substantial homogeneity in baseline characteristics (age, sex, comorbidities, tobacco use and COPD characteristics) across the two subgroups, that were also balanced in size, increasing the internal validity of our findings.

The outcomes of critically ill AECOPD patients are based not only on the respiratory failure severity, but also on the magnitude of the systemic inflammatory response. Various respiratory parameters such as P_a_O_2_, P_a_CO_2_, oxygenation index and the need for NIV or IMV are widely integrated into composite prognostic scores for in-hospital mortality or the need for ICU admission in the AECOPD population [25,26]. However, these models are less consistent for predicting ICU mortality specifically, and they may not fully reflect the clinical severity of systemic inflammation and organ dysfunction that defines the critically ill patient. Notably, they do not account for AECOPD etiology and the different pathophysiological mechanisms associated with infectious and non-infectious exacerbations. Taken together, this highlights the clinical relevance of identifying novel prognostic markers in these subgroups.

Derived hematological indices of inflammation like NLR, PLR, MLR, SII, and SIRI were widely analyzed across different pathologies and in diverse clinical settings [27,28,29,30,31,32,33]. Their integration into current clinical practice is supported by their prognostic value in a broad spectrum of inflammatory states [18,24]. In patients with stable COPD, indices such as NLR, MLR and PLR can be chronically elevated [24,34,35,36]. More importantly, higher NLR and PLR values were associated with increased risk of exacerbations over one year and higher in-hospital death risk [24,34,37]. Elevated NLR values were independently associated with the presence of pulmonary hypertension in COPD patients, stating that NLR may represent an auxiliary diagnostic indicator in this population [38]. NLR, PLR and SIRI could be used as early indicators of pulmonary hypertension in patients with AECOPD, with NLR ≥ 4.659 predicting echocardiographic pulmonary hypertension in one AECOPD cohort (sensitivity 81.2%, specificity 59.5%, AUC 0.701) [39].

In our cohort, marked differences were observed between the two studied subgroups. Infectious AECOPD demonstrated a distinct inflammatory profile, with higher leukocyte and neutrophil counts, elevated CRP and procalcitonin levels, and lower lymphocyte and eosinophil counts. Moreover, this enhanced inflammatory response translated into increased clinical severity. Patients with infectious AECOPD showed more organ dysfunction and physiological disturbances as reflected by higher SOFA and APACHE II scores, more severe respiratory failure as reflected by more frequent need for IMV during LOS, longer duration of IMV and higher need for NMBA. Overall, these findings suggest that the infectious etiology, through its maladaptive inflammatory pathways, could be a determinant of the higher disease burden and eventually, worse outcomes including increased mortality, as in our study [11].

Therefore, derived hematological indices such as NLR, dNLR, NPR, SII, SIRI, PLR and MLR could more comprehensively capture the systemic inflammatory response and serve as surrogate prognostic markers according to AECOPD etiology. In our cohort, ROC analysis showed that several derived hematological indices had fair to very good discriminatory performance for mortality prediction in non-infectious AECOPD, specifically NPR, SII, MLR and SIRI, with AUROCs ranging from 0.7 to 0.855. After adjustment for confounding factors, SIRI, SII and monocytes remained independent predictors. In contrast, for infectious AECOPD, only NPR retained its independent predictive value; its discriminatory capacity improved further when the analysis was restricted to bacterial AECOPD.

The immunological and molecular mechanisms underlying these differences remain incompletely elucidated in the available literature. Bacterial exacerbations are typically characterized by a predominant neutrophilic inflammatory response driven by pathogen-induced cytokine release (tumor necrosis factor-alpha and interleukin 6) and enhanced neutrophil chemotaxis [11,40]. Consistent with this, plasma heparan sulphate—a mediator known to facilitate neutrophil migration to the site of injury—has been shown to correlate positively with an infectious AECOPD etiology [41]. In contrast, non-infectious AECOPD involves a substantially attenuated neutrophilic response, decreased macrophage cytokine expression and phagocytosis activity [7,11], thus magnifying the relative changes in platelet activation and/or the shifts in monocyte/lymphocyte balance. This could explain the enhanced prognostic performance of composite hematological indices (i.e., MLR and SIRI) in this latter subgroup.

Lastly, NLR—a widely studied, highly cited, but still conflicting index [42,43]—did not demonstrate an adequate predictive performance for mortality in either of the studied subgroups. Although this marker was associated with several adverse outcomes in stable COPD and AECOPD [44], our findings suggest that it cannot specifically discriminate prognostic differences according to AECOPD etiology. This divergence highlights the importance of validating composite markers able not only to predict adverse outcomes, but also to reflect pathological differences underlying distinct AECOPD etiologies. A better understanding of these mechanisms could facilitate the development of individualized therapies in AECOPD, including targeted immunotherapy [10].

Our study is subject to several limitations. Firstly, it is a retrospective, monocentric study which increases the risk of selection and geographical bias, including the risk of a wrong classification into infectious or non-infectious AECOPD. Although the frequency of ICU admission was in line with published data and we analyzed a particular population of AECOPD patients, our sample size was only 88 patients, which warrants caution in results interpretation, especially because we conducted subgroup analyses which may further reduce statistical power. Moreover, the retrospective design precludes us from establishing a causal relationship between hematologic indices and outcomes; thus, our findings remain hypothesis-generating. We did not collect data regarding several potential confounding factors, i.e., chronic steroid therapy, the time since steroid administration in hospital and the complete blood count considered for analysis, fluid resuscitation, and blood transfusions. Furthermore, interleukin measurement was not performed in any patient, while other biomarkers had a significant proportion of missing values (e.g., D-dimers, C-reactive protein, etc.). Lastly, hematologic indices were derived from a single measurement, while dynamic hematological indices could show enhanced discriminative power [28].

## 5. Conclusions

This study provides novel insights by differentiating prognostic factors for ICU mortality in AECOPD patients based on etiology. Derived hematological indices demonstrated variable predictive power in different subgroups: non-infectious, infectious and bacterial AECOPD. MLR and SIRI showed very good predictive power for ICU mortality in non-infectious AECOPD. NPR was the only index that demonstrated fair predictive power in infectious AECOPD, which improved to moderate in bacterial AECOPD. Against this background, this study highlights the importance of individualized prognostication in AECOPD.

## Figures and Tables

**Table 1:** Characteristics of the study population at ICU admission.

Parameter	Total Sample AECOPD (n = 88)	Non-Infectious AECOPD (n = 40)	Infectious AECOPD (n = 48)	*p* Value
Gender, Male, n (%)	59 (67%)	26 (65%)	33 (68.8%)	0.709
Age, mean (±SD)	66.78 (±10.67)	67.4 (9.83)	66.25 (11.4)	0.61
Obesity, n (%)	32 (36.4%)	18 (45%)	14 (29.2%)	0.124
Arterial hypertension, n (%)	68 (77.3%)	31 (77.5%)	37 (77.1%)	0.963
Left heart failure, n (%)	32 (36.4%)	14 (35%)	18 (37.5%)	0.808
Atrial fibrillation, n (%)	24 (27.3%)	9 (22.5%)	15 (31.3%)	0.359
Diabetes mellitus, n (%)	29 (33%)	13 (32.5%)	16 (33.3%)	0.934
Chronic kidney disease, n (%)	8 (9.1%)	2 (5%)	6 (12.5%)	0.283
Cirrhosis, n (%)	3 (3.4%)	0 (0%)	3 (6.3%)	0.108
Stroke, n (%)	10 (11.4%)	4 (10%)	6 (12.5%)	0.75
GERD, n (%)	2 (2.3%)	2 (5%)	0 (0%)	0.204
Oncology, n (%)	3 (3.4%)	2 (5%)	1 (2.1%)	0.589
Alcoholism, n (%)	16 (18.2%)	9 (22.5%)	7 (14.6%)	0.338
**Respiratory comorbidities**				
Chronic cor pulmonale, n (%)	48 (54.5%)	22 (55%)	26 (54.2%)	0.938
Pulmonary hypertension, n (%)	54 (61.4%)	24 (60%)	30 (62.5%)	0.810
OSA, n (%)	7 (8%)	3 (7.5%)	4 (8.3%)	1
OHS, n (%)	16 (18.2%)	10 (25%)	6 (12.5%)	0.130
Asthma, n (%)	8 (8.1%)	3 (7.5%)	5 (10.4%)	0.723
PE history, n (%)	1 (1.1%)	1 (2.5%)	0 (0%)	0.455
Tuberculosis history, n (%)	18 (20.5%)	8 (20%)	10 (20.8%)	0.923
Smoker, n (%)	78 (88.6%)	34 (85%)	44 (91.7%)	0.326
Smoker packs/year	40 [20–50]	40 [20–57.5]	40 [20–50]	0.577
Professional exposure, n (%)	8 (9.1%)	5 (12.5%)	3 (6.3%)	0.46
**COPD characteristics**				
GOLD stage 2, n (%)	4 (4.5%)	3 (7.5%)	1 (2%)	0.148 0.058
GOLD stage 3, n (%)	13 (14.8%)	5 (12.5)	8 (16.7%)	
GOLD stage 4, n (%)	52 (59.1%)	27 (67.5%)	25 (52.1%)	
GOLD stage unknown, n (%)	19 (21.6%)	5 (12.5%)	14 (29.2%)	
Home oxygen therapy, n (%)	40 (45.5%)	19 (47.5%)	21 (43.8%)	0.725
Home NIV, n (%)	5 (5.7%)	3 (7.5%)	2 (4.2%)	0.656
Number of exacerbations/year				
0, n (%)	41 (46.6%)	22 (55%)	19 (39.6%)	0.28
1, n (%)	2 (2.3%)	1 (2.5%)	1 (2.1%)	
2, n (%)	15 (17%)	7 (17.5%)	8 (16.7%)	
>2, n (%)	15 (17%)	7 (17.5%)	8 (16.7%)	
Unknown, n (%)	15 (17%)	3 (7.5%)	12 (25%)	0.058
Charlson Comorbidity Index, median [IQR]	4 [3–6]	4 [3.25–6]	4.5 [3–6]	0.932
**Respiratory support, adjuvants and pharmacotherapy at ICU admission and during ICU LOS**				
IMV, n (%)	28 (31.8%)	12 (30%)	16 (33.3%)	0.738
NIV, n (%)	60 (68.2%)	28 (70%)	32 (66.7%)	0.738
Intubated during LOS, n (%)	36 (60%)	13 (46.4%)	23 (72%)	0.045
Hours of IMV, median [IQR]	49 [0–200]	15.5 [0–183]	111 [33–213]	0.019
Hours of NIV, median [IQR]	4 [0–41.25]	21 [0–59]	1.5 [0–19]	0.015
Hypnotics, n (%)	59 (67%)	23 (57.5%)	36 (75%)	0.082
Opioids, n (%)	49 (55.7%)	19 (47.5%)	30 (62.5%)	0.158
NMBA, n (%)	28 (31.8%)	7 (17.5%)	21 (43.8%)	0.008
Bronchodilators, n (%)	88 (100%)	40 (100%)	48 (100%)	1
Systemic corticosteroids, n (%)	81 (92%)	37 (92.5%)	44 (91.7%)	0.866
Vasopressor requirement, n (%)	49 (55.7%)	13 (32.5%)	36 (75%)	<0.001
**Severity and risk scores**				
SOFA median [IQR]	6 [3.25–9]	5 [3–7.5]	7 [4–9.75]	0.034
APACHE II median [IQR]	19 [14–26]	18 [14–22]	20.5 [16–31]	0.046
**Laboratory parameters**				
Leukocytes (×10^3^/mm^3^), median [IQR]	12.94 [8.5–17.1]	11.4 [7.7–13.9]	15.1 [10.6–19.6]	0.002
Neutrophils (×10^3^/mm^3^), median [IQR]	10.64 [7.1–15.1]	8.62 [6.15–11.73]	13.97 [8.87–17.42]	<0.001
Lymphocytes (×10^3^/mm^3^), median [IQR]	0.72 [0.5–1.04]	0.87 [0.69–1.27]	0.57 [0.44–0.87]	<0.001
Monocytes (×10^3^/mm^3^), median [IQR]	0.69 [0.37–1.04]	0.75 [0.36–1]	0.62 [0.41–1.14]	0.987
Eosinophils (×10^3^/mm^3^), median [IQR]	0.01 [0–0.04]	0.01 [0–0.058]	0 [0–0.018]	0.041
Basophils (×10^3^/mm^3^), median [IQR]	0.02 [0.01–0.048]	0.02 [0.13–0.058]	0.03 [0.01–0.04]	0.905
Platelets (×10^3^/mm^3^), median [IQR]	209 [164–266]	197 [157–244]	228 [165–283]	0.241
dNLR, median [IQR]	7.04 [4.23–10.95]	5.26 [3.47–7.05]	9.57 [6.12–14.73]	<0.001
NLR, median [IQR]	14.3 [8.42–24.98]	8.58 [6.34–13.5]	19.9 [13.57–31.92]	<0.001
NPR, median [IQR]	0.046 [0.036–0.072]	0.044 [0.035–0.053]	0.059 [0.038–0.079]	0.023
SII, median [IQR]	3303 [1545–5866]	1605 [957–3177]	4559 [2464–6796]	<0.001
PLR, median [IQR]	288 [160–422]	190 [145–344]	364 [211–467]	<0.001
MLR, median [IQR]	0.79 [0.44–1.37]	0.61 [0.38–0.97]	1.02 [0.58–1.6]	0.01
SIRI, median [IQR]	9.06 [3.29–18.77]	5.16 [2.93–11.11]	15.18 [5.22–24.95]	<0.001
Erythrocytes (×10^6^/mm^3^) (±SD)	4.5 (±0.92)	4.65 (±0.93)	4.39 (0.9±)	0.183
Hemoglobin (g/dL), mean (±SD)	12.76 (±2.63)	13.34 (±2.87)	12.27 (±2.33)	0.059
Haematocrit %, mean (±SD)	41.3 (±9.19)	43.3 (±9.6)	39.64 (±8.57)	0.062
MCV (fL), mean (±SD)	91.54 (±8)	94.04 (±8.98)	90.28 (±6.94)	0.108
MCH (pg), mean (±SD)	28.42 (±3.15)	28.77 (±3.74)	28.13 (±2.56)	0.343
MCHC (g/dL), mean (±SD)	30.95 [29.6–32.1]	30.56 [29.3–31.5]	31.5 [29.8–32.5]	0.11
RDW (%), median [IQR]	14.4 [13.5–16.6]	14 [13.3–16.5]	14.7 [13.8–16.8]	0.167
Erythroblasts/mm^3^, median [IQR]	0.01 [0–0.02]	0.01 [0–0.02]	0.01 [0–0.035]	0.554
Sodium (mmol/L), median [IQR]	138 [134–141]	138 [135–140]	138 [133–142]	0.84
Creatinine (mg/dL), median [IQR]	0.9 [0.7–1.36]	0.83 [0.72–1.28]	1.1 [0.66–1.44]	0.475
Urea (mg/dL), median [IQR]	56 [38–80]	55 [34–64]	60 [42.7–94]	0.137
Bilirubin (mg/dL), median [IQR]	0.9 [0.5–1.15]	0.93 [0.53–1.27]	0.83 [0.45–1.08]	0.404
P_a_CO_2_ (mmHg), mean (±SD)	86 (±25.42)	88.83 (±24.55)	83.55 (±26.13)	0.335
P_a_O_2_/FiO_2_ ratio, median [IQR]	180 [124–266]	195 [124–326]	170 [117–236]	0.06
Lactate (mmol/L), median [IQR]	1.21 [0.98–2.23]	1.1 [0.92–1.64]	1.3 [1–2.58]	0.199
Procalcitonin (ng/mL), median [IQR] (n = 77)	0.48 [0.12–1.9]	0.18 [0.09–0.5]	1.27 [0.28–4.5]	<0.001
CRP (n = 51), median [IQR]	45 [19–125]	16.1 [8–28.5]	86 [46.5–241.5]	<0.001
Fibrinogen (mg/dL), median [IQR]	418 [351–541]	385 [334–437]	465 [374–577]	0.009
D-dimers (mg/dL), median [IQR], (n = 43)	644 [356–1190]	590 [303–816]	688 [392–1323]	0.5
**ICU-related outcomes and length of stay**				
Tracheostomy, n (%)	8 (9.1%)	4 (10%)	4 (8.3%)	1
VAP, n (%)	32 (36.4%)	14 (35%)	18 (37.5%)	0.808
New AKI, n (%)	38 (43.2%)	12 (32.5%)	25 (52.1%)	0.065
LOS-ICU, median [IQR]	5 [3–12]	5 [2–9.75]	6 [3–12]	0.213
LOS-Hospital, median [IQR]	11.5 [7–18]	11 [7.25–20]	11.5 [7–18]	0.554
ICU mortality, n (%)	39	8 (20%)	31 (64.6%)	<0.001
Hospital mortality, n (%)	43 (48.9%)	11 (27.5%)	32 (66.7%)	<0.001

Comparisons between categorical variables were conducted using the Chi-square test or Fisher Exact (for cells with less than five observations); comparisons between continuous variables from two independent groups were conducted using the Independent *t*-test or the Mann–Whitney U test for parametric and non-parametric distribution, respectively. Abbreviations: AECOPD = Acute Exacerbation of Chronic Obstructive Pulmonary Disease; AKI = Acute Kidney Injury; APACHE = Acute Physiology And Chronic Health Evaluation; AUC = Area Under the Curve, CCI = Charlson Comorbidity Index; CI = Confidence Interval; CRP = C-reactive Protein; dNLR = derived Neutrophil-to-Lymphocyte Ratio; GERD = Gastro-Esophageal Reflux Disease; GOLD = Global Initiative for Chronic Obstructive Lung Disease; ICU = Intensive Care Unit; IMV = Invasive Mechanical Ventilation; LOS = Length of Stay; MCH = Mean Corpuscular Hemoglobin; MCHC = Mean Corpuscular Hemoglobin Concentration; MCV = Mean Corpuscular Volume; MLR = Monocyte-to-Lymphocyte ratio; n = number; NIV = Non-invasive Ventilation; NLR = Neutrophil-to-Lymphocyte Ratio; NMBA = Neuromuscular Blocking Agents; NPR = Neutrophil-to-Platelet Ratio; OHS = Obesity Hypoventilation Syndrome; OSA = Obstructive Sleep Apnea; P_a_CO_2_ = carbon dioxide partial pressure in arterial blood; P_a_O_2_/FiO_2_ ratio = ratio between partial pressure of oxygen in arterial blood and inspired fraction of oxygen; PE = Pulmonary Embolism; PLR = Platelet-to-Lymphocyte ratio; RDW = Red Blood Cell Distribution Width; SD = Standard Deviation; SII = Systemic Inflammation Index; SIRI = Systemic Inflammation Response Index; SOFA = Sequential Organ Failure Assessment; VAP = Ventilator-Associated Pneumonia.

**Table 2:** ROC analysis for the hematological and non-hematological predictors.

Parameter	All AECOPD AUC (n = 88)	95% CI	*p* Value	Non- Infectious AECOPD AUC (n = 40)	95% CI	*p* Value	Infectious AECOPDAUC (n = 48)	95% CI	*p* Value	Bacterial AECOPDAUC (n = 40)	95% CI	*p* Value
**Hematological predictors**												
Leukocytes	0.696	0.58–0.81	0.001	0.750	0.59–0.91	0.002	0.607	0.45–0.77	0.189	0.693	0.53–0.86	0.023
Neutrophils	0.710	0.6–0.82	0.000	0.766	0.61–0.92	0.001	0.613	0.45–0.77	0.165	0.697	0.53–0.86	0.020
Lymphocytes	0.595	0.48–0.71	0.120	0.51	0.3–0.72	0.929	0.506	0.33–0.68	0.948	0.51	0.32–0.7	0.954
Monocytes	0.613	0.49–0.73	0.064	0.791	0.63–0.95	<0.001	0.555	0.39–0.72	0.519	0.591	0.41–0.77	0.315
Eosinophils	0.538	0.41–0.66	0.547	0.453	0.25–0.66	0.656	0.535	0.37–0.7	0.680	0.593	0.42–0.77	0.299
Basophils	0.555	0.44–0.68	0.372	0.533	0.34–0.73	0.740	0.594	0.42–0.77	0.282	0.643	0.46–0.82	0.124
Platelets	0.536	0.41–0.66	0.569	0.563	0.37–0.76	0.530	0.70	0.51–0.82	0.032	0.68	0.51–0.85	0.033
dNLR	0.654	0.53–0.77	0.013	0.602	0.37–0.84	0.397	0.537	0.37–0.71	0.665	0.624	0.45–0.8	0.168
NLR	0.718	0.61–0.83	<0.001	0.703	0.48–0.93	0.078	0.569	0.41–0.73	0.408	0.635	0.46–0.81	0.134
NPR	0.707	0.59–0.82	<0.001	0.703	0.52–0.89	0.029	0.691	0.54–0.84	0.012	0.781	0.64–0.93	<0.001
SII	0.659	0.54–0.78	0.007	0.715	0.5–0.93	0.049	0.452	0.28–0.62	0.574	0.483	0.3–0.67	0.857
PLR	0.553	0.43–0.67	0.395	0.609	0.39–0.83	0.319	0.658	0.49–0.82	0.060	0.664	0.49–0.84	0.063
MLR	0.693	0.58–0.81	0.001	0.820	0.67–0.97	<0.001	0.564	0.4–0.73	0.551	0.576	0.25–0.6	0.399
SIRI	0.732	0.62–0.84	<0.001	0.855	0.71–1	<0.001	0.607	0.45–0.77	0.191	0.664	0.5–0.83	0.057
Hemoglobin	0.612	0.49–0.79	0.063	0.531	0.32–0.75	0.776	0.615	0.45–0.77	0.167	0.585	0.41–0.76	0.345
Haematocrit	0.628	0.51–0.75	0.034	0.531	0.32–0.74	0.768	0.654	0.5–0.81	0.054	0.616	0.44–0.79	0.191
MCV	0.6	0.48–0.72	0.099	0.588	0.39–0.78	0.376	0.564	0.4–0.73	0.449	0.563	0.38–0.75	0.505
MCH	0.501	0.38–0.62	0.983	0.545	0.31–0.77	0.702	0.597	0.43–0.77	0.260	0.556	0.37–0.75	0.564
MCHC	0.665	0.55–0.78	0.006	0.512	0.28–0.75	0.922	0.734	0.59–0.88	0.001	0.676	0.51–0.85	0.042
RDW%	0.557	0.44–0.68	0.355	0.531	0.31–0.75	0.780	0.512	0.34–0.69	0.891	0.551	0.36–0.74	0.598
Erythroblasts	0.597	0.48–0.72	0.115	0.625	0.4–0.85	0.278	0.585	0.42–0.75	0.320	0.656	0.48–0.83	0.080
Fibrinogen	0.53	0.4–0.65	0.66	0.44	0.22–0.66	0.59	0.45	0.28–0.62	0.54	0.44	0.26–0.63	0.55
**Non-hematological predictors**												
CCI	0.617	0.5–0.74	0.057	0.646	0.41–0.88	0.216	0.640	0.48–0.8	0.093	0.583	0.4–0.77	0.380
GCS	0.604	0.48–0.73	0.098	0.746	0.56–0.93	0.011	0.593	0.43–0.75	0.260	0.593	0.42–0.77	0.301
SOFA	0.771	0.67–0.87	0.000	0.689	0.47–0.91	0.091	0.813	0.69–0.93	<0.001	0.855	0.74–0.97	<0.001
APACHE II	0.720	0.62–0.83	0.000	0.680	0.48–0.88	0.084	0.720	0.57–0.87	0.004	0.761	0.61–0.91	0.001
Sodium	0.569	0.44–0.7	0.288	0.506	0.25–0.76	0.964	0.617	0.46–0.78	0.155	0.613	0.44–0.79	0.208
Creatinine	0.611	0.49–0.73	0.072	0.588	0.4–0.78	0.371	0.633	0.47–0.79	0.107	0.605	0.43–0.78	0.247
Urea	0.661	0.55–0.77	0.005	0.619	0.43–0.81	0.210	0.664	0.49–0.84	0.063	0.648	0.46–0.84	0.124
Bilirubin	0.522	0.4–0.65	0.729	0.518	0.26–0.78	0.895	0.51	0.34–0.68	0.907	0.505	0.32–0.69	0.955
P_a_CO_2_	0.579	0.49–0.74	0.211	0.568	0.34–0.8	0.565	0.662	0.51–0.82	0.04	0.56	0.38–0.74	0.515
P_a_O_2_/FiO_2_ ratio	0.583	0.46–0.71	0.183	0.547	0.3–0.8	0.712	0.534	0.37–0.7	0.682	0.516	0.6–0.91	0.863
Lactate	0.724	0.62–0.83	<0.001	0.68	0.48–0.89	0.086	0.769	0.63–0.91	0.000	0.755	0.34–0.7	0.001

Abbreviations: AECOPD = Acute Exacerbation of Chronic Obstructive Pulmonary Disease; APACHE = Acute Physiology And Chronic Health Evaluation; AUC = Area Under the Curve, CCI = Charlson Comorbidity Index; CI = Confidence Interval; dNLR = derived Neutrophil-to-Lymphocyte Ratio; GCS = Glasgow Coma Scale; MCH = Mean Corpuscular Hemoglobin; MCHC = Mean Corpuscular Hemoglobin Concentration; MCV = Mean Corpuscular Volume; MLR = Monocyte-to-Lymphocyte ratio; NLR = Neutrophil-to-Lymphocyte Ratio; NPR = Neutrophil-to-Platelet Ratio; PLR = Platelet-to-Lymphocyte ratio; RDW = Red Blood Cell Distribution Width; SII = Systemic Inflammation Index; SIRI = Systemic Inflammation Response Index; SOFA = Sequential Organ Failure Assessment; P_a_CO_2_ = carbon dioxide partial pressure in arterial blood; P_a_O_2_/FiO_2_ ratio = ratio between partial pressure of oxygen in arterial blood and inspired fraction of oxygen.

**Table 3:** Sensitivity, specificity and likelihood ratios for the identified cut-off values.

All Sample AECOPD						
Parameter	Cut-off 95% CI	Youden index	Sensitivity 95% CI	Specificity 95% CI	+LR 95% CI	−LR 95% CI
Leukocytes	>14.04 10.21–19.82	0.324 0.136–0.428	58.97 42.1–74.4	73.47 58.9–85.1	2.22 1.30–3.79	0.56 0.37–0.84
Neutrophils	>13.45 11.38–17.61	0.38 0.192–0.513	56.41 39.6–72.2	81.63 68.0–91.2	3.07 1.60–5.89	0.53 0.36–0.78
dNLR	>10.53 8.39–14.59	0.313 0.147–0.422	43.6 27.8–60.4	87.76 75.2–95.4	3.56 1.55–8.17	0.64 0.48–0.86
NLR	>10.12 4.1–16.5	0.382 0.202–0.474	87.18 72.6–95.7	51 36.3–65.6	1.78 1.31–2.43	0.25 0.11–0.60
NPR	>0.061 0.035–0.066	0.4 0.21–0.53	56.41 39.6–72.2	83.67 70.3–92.7	3.46 1.73–6.90	0.52 0.36–0.76
SII	>3459 1627–5950	0.294 0.103–0.4	64.1 47.2–78.8	65.31 50.4–78.3	1.85 1.18–2.90	0.55 0.34–0.88
MLR	>1.054 0.543–1.92	0.314 0.133–0.4	53.85 37.2–69.9	77.55 63.4–88.2	2.4 1.32–4.35	0.6 0.41–0.86
SIRI	>15.27 10.67–34.47	0.421 0.238–0.55	56.41 39.6–72.2	85.71 72.8–94.1	3.95 1.89–8.27	0.51 0.35–0.74
Haematocrit	<43.5 41.7–53.1	0.3 0.14–0.45	76.92 60.7–88.9	53.1 38.3–67.5	1.64 1.16–2.31	0.43 0.23–0.82
MCHC	>31.55 31.1–32.1	0.38 0.181–0.54	56.41 39.6–72.2	81.63 68.0–91.2	3.07 1.60–5.89	0.53 0.36–0.78
Non-infectious AECOPD						
Parameter	Cut-off 95% CI	Youden index	Sensitivity 95% CI	Specificity 95% CI	+LR 95% CI	−LR 95% CI
Leukocytes	>10.32 9.31–15.44	0.5313 0.344–0.688	100% 63.1–100.0	53.13 34.7–70.9	2.13 1.48–3.08	0.00
Neutrophils	>8.075 7.82–16.85	0.563 0.375–0.719	100% 63.1–100.0	56.25 37.7–73.6	2.29 1.54–3.39	0.00
Monocytes	>1 0.75–1.5	0.5 0.313–0.625	62.5 24.5–91.5	87.5 71.0–96.5	5.00 1.73–14.47	0.43 0.17–1.06
NPR	>0.0397 0.0341–0.05	0.406 0.221–0.531	100% 63.1–100.0	40.63 23.7–59.4	1.47 1.00–2.17	
SII	>4588 1951–7088	0.438 0.188–0.688	50 15.7–84.3	93.75 79.2–99.2	8 1.77–36.22	0.53 0.27–1.07
MLR	>0.543 0.429–0.976	0.531 0.344–0.656	100% 63.1–100.0	53.13 34.7–70.9	2.13 1.48–3.08	
SIRI	>10.96 4.12–18.77	0.625 0.365–0.813	75 34.9–96.8	87.5 71.0–96.5	6.00 2.21–16.31	0.29 0.085–0.96
Infectious AECOPD						
Parameter	Cut-off 95% CI	Youden index	Sensitivity 95% CI	Specificity 95% CI	+LR 95% CI	−LR 95% CI
Platelets	<209 126–321	0.287 0.13–0.39	58.06 39.1–75.5	70.59 44.0–89.7	1.97 0.89–4.37	0.59 0.35–0.99
NPR	>0.0657 0.064–0.096	0.45 0.233–0.647	61.3 42.2–78.2	82.35 56.6–96.2	3.47 1.2–10.07	0.47 0.29–0.77
MCHC	>31.5 31.1–34.9	0.433 0.199–0.644	64.52 43.9–80.1	88.24 63.6–98.5	5.48 1.45–20.69	0.4 0.24–0.67
Bacterial AECOPD						
Parameter	Cut-off 95% CI	Youden index	Sensitivity 95% CI	Specificity 95% CI	+LR 95% CI	−LR 95% CI
Leukocytes	>11.08 6.44–17.93	0.347 0.133–0.493	88 68.8–97.5	46.67 21.3–73.4	1.65 1.01–2.71	0.26 0.078–0.85
Neutrophils	>13.58 9.48–17.61	0.36 0.16–0.53	76 54.9–90.6	60 32.3–83.7	1.9 0.98–3.67	0.4 0.18–0.90
Platelets	<154 96–207	0.32 0.16–0.44	32 14.9–53.5	100 78.2–100.0		0.68 0.52–0.89
NPR	>0.0657 0.043–0.096	0.52 0.28–0.723	72 50.6–87.9	80 51.9–95.7	3.6 1.27–10.20	0.35 0.18–0.69
MCHC	>31.55 31.1–33	0.467 0.21–0.69	60 38.7–78.9	88.67 59.5–98.3	4.5 1.19–17.00	0.45 0.27–0.78

Cut-off values were identified using the Youden Index. The corresponding sensitivity, specificity and likelihood ratios are reported. Abbreviations: AECOPD = Acute Exacerbation of Chronic Obstructive Pulmonary Disease; CI = Confidence Interval; dNLR = derived Neutrophil-to-Lymphocyte Ratio; +LR = Positive Likelihood Ratio; −LR = Negative Likelihood Ratio; MCHC = Mean Corpuscular Hemoglobin Concentration; MLR = Monocyte-to-Lymphocyte ratio; NLR = Neutrophil-to-Lymphocyte Ratio; NPR = Neutrophil-to-Platelet Ratio; PLR = Platelet-to-Lymphocyte ratio; SII = Systemic Inflammation Index; SIRI = Systemic Inflammation Response Index.

**Table 4:** Kaplan–Meier Analysis of ICU mortality for the Studied Hematological Parameters.

All Sample AECOPD					
Parameter	Cut-off	HR	95% CI	Χ^2^-value	*p* value
Leukocytes	>14.04	2.03	1.05–3.9	4.44	0.035
Neutrophils	>13.45	2.34	1.2–4.57	6.17	0.013
dNLR	>10.53	2.87	1.35–6.09	7.55	0.006
NLR	>10.12	2.14	1.03–4.44	4.15	0.04
NPR	>0.061	2.88	1.45–5.7	9.16	0.003
SII	>3459	1.74	0.9–3.35	2.72	0.099
MLR	>1.054	1.5	0.78–2.89	1.49	0.22
SIRI	>15.27	2.26	1.15–4.4	5.66	0.017
Haematocrit	<43.5	2.26	1.18–4.35	6	0.014
MCHC	>31.55	3.19	1.58–6.43	10.53	0.001
Non-infectious AECOPD					
Parameter	Cut-off	HR	95% CI	Χ^2^-value	*p* value
Leukocytes	>10.32	-	-	4.42	0.035
Neutrophils	>8.075	-	-	4.57	0.03
Monocytes	>1	2.98	0.67–13.19	2.075	0.15
NPR	>0.0397	-	-	3.08	0.08
SII	>4588	1219.9	72.6–20,503	24.36	<0.0001
MLR	>0.543	-	-	3.11	0.078
SIRI	>10.96	9.7	2.06–45.74	8.28	0.004
Infectious AECOPD					
Parameter	Cut-off	HR	95% CI	Χ^2^-value	*p* value
Platelets	<209	2.39	1.1–5.21	4.85	0.027
NPR	>0.0657	1.94	0.9–4.19	2.88	0.09
MCHC	>31.5	3.22	1.48–7.02	8.7	0.003
Bacterial AECOPD					
Parameter	Cut-off	HR	95% CI	Χ^2^-value	*p* value
Leukocytes	>11.08	1.36	0.4–4.55	0.25	0.62
Neutrophils	>13.58	1.65	0.68–3.96	1.19	0.27
Platelets	<154	7.43	2.1–26.3	9.68	0.002
NPR	>0.0657	2.57	1.1–6.04	4.7	0.029
MCHC	>31.55	3.35	1.4–8.13	7.17	0.007

In all models the outcome studied was ICU mortality during ICU length of stay. Each hematological parameter was dichotomic (based on the identified cut-off value) and was introduced as the “Factor” in the analysis. If a hazard ratio value is not reported, this is because all cases from one subgroup were censored as the studied event (ICU mortality) did not occur in any patient. Abbreviations: AECOPD = Acute Exacerbation of Chronic Obstructive Pulmonary Disease; CI = Confidence Interval; dNLR = derived Neutrophil-to-Lymphocyte Ratio; ICU = Intensive Care Unit; MCHC = Mean Corpuscular Hemoglobin Concentration; MLR = Monocyte-to-Lymphocyte ratio; NLR = Neutrophil-to-Lymphocyte Ratio; NPR = Neutrophil-to-Platelet Ratio; SII = Systemic Inflammation Index; SIRI = Systemic Inflammation Response Index.

## Data Availability

The original contributions presented in this study are included in the article/Supplementary Materials. Further inquiries can be directed to the corresponding author.

## Supplementary Materials

The following supporting information can be downloaded at: https://www.mdpi.com/article/10.3390/medicina62040728/s1, Table S1: STROBE Checklist. Table S2: Multivariable binary regression model for leukocytes. Table S3: Multivariable binary regression model for neutrophils. Table S4: Multivariable binary regression model for dNLR. Table S5: Multivariable binary regression model for NLR. Table S6: Multivariable binary regression model for NPR. Table S7: Multivariable binary regression model for SII. Table S8: Multivariable binary regression model for MLR. Table S9: Multivariable binary regression model for SIRI. Table S10: Multivariable binary regression model for haematocrit. Table S11: Multivariable binary regression model for MCHC. Table S12: Multivariable binary regression model for SIRI. Table S13: Multivariable binary regression model for SII. Table S14: Multivariable binary regression model for monocytes. Table S15: Multivariable binary regression model for platelets. Table S16: Multivariable binary regression model for MCHC. Table S17: Multivariable binary regression model for NPR. Table S18: Multivariable binary regression model for leukocytes. Table S19: Multivariable binary regression model for neutrophils. Table S20: Multivariable binary regression model for NPR. Table S21: Multivariable binary regression model for MCHC.

## Abbreviations

The following abbreviations are used in this manuscript:

AECOPD	Acute Exacerbation of Chronic Obstructive Pulmonary Disease
AKI	Acute Kidney Injury
APACHE	Acute Physiology And Chronic Health Evaluation
AUC	Area Under the Curve
CCI	Charlson Comorbidity Index
CI	Confidence Interval
CRP	C-reactive Protein
dNLR	derived Neutrophil-to-Lymphocyte Ratio
GCS	Glasgow Coma Scale
GERD	Gastro-Esophageal Reflux Disease
GOLD	Global Initiative for Chronic Obstructive Lung Disease
HR	Hazard Ratio
ICD-10	International Classification of Diseases 10th edition
ICU	Intensive Care Unit
IMV	Invasive Mechanical Ventilation
IQR	Interquartile Range
LOS	Length of Stay
+LR	Positive Likelihood Ratio
−LR	Negative Likelihood Ratio
MCH	Mean Corpuscular Hemoglobin
MCHC	Mean Corpuscular Hemoglobin Concentration
MCV	Mean Corpuscular Volume
MLR	Monocyte-to-Lymphocyte ratio
NIV	Non-invasive Ventilation
NLR	Neutrophil-to-Lymphocyte Ratio
NMBA	Neuromuscular Blocking Agents
NPR	Neutrophil-to-Platelet Ratio
OHS	Obesity Hypoventilation Syndrome
OSA	Obstructive Sleep Apnea
PE	Pulmonary Embolism
PLR	Platelet-to-Lymphocyte ratio
RDW	Red Blood Cell Distribution Width
ROC	Receiver Operating Characteristics
P_a_CO_2_	Carbon Dioxide Partial Pressure in Arterial Blood
P_a_O_2_/FiO_2_	Ratio Between Partial Pressure of Oxygen in Arterial Blood and Inspired Fraction of Oxygen
SD	Standard Deviation
SII	Systemic Inflammation Index
SIRI	Systemic Inflammation Response Index
SOFA	Sequential Organ Failure Assessment
SPSS	Statistical Package for Social Sciences
STROBE	Strengthening the Reporting of Observational Studies in Epidemiology
VAP	Ventilator-Associated Pneumonia
