# Supplementary material for: One Index Does Not Predict All—Hematological Derived Indices Have Different Predictive Value for ICU Mortality in Critically Ill Patients with Non-Infectious Versus Infectious Acute Exacerbation of COPD
Source: Medicina (Kaunas). 2026 Apr 10;62(4):728. doi: 10.3390/medicina62040728 (PMC13117956; doi:10.3390/medicina62040728)
Supplement: Supplementary file 1 [file medicina-62-00728-s001.zip › Supplementary Materials S2.pdf]

Supplementary Materials S2. Multivariable binary regressions to test the independent predictive value of the hematological parameters studied.

1. **Multivariable binary regressions for the studied hematological parameters in all AECOPD patients**

Table S2 Multivariable binary regression model for leukocytes.

All AECOPD, Method: Enter, Nagelkerke R^2: 0.585, Hosmer–Lemeshow test p = 0.62, Overall prediction: 81.8%								
							95% C.I.for EXP(B)	
	B	S.E.	Wald	df	*p* value	Exp(B)	Lower	Upper
Leukocytes >14.04	1.090	0.636	2.943	1	0.086	2.975	0.856	10.339
Gender	0.793	0.706	1.260	1	0.262	2.209	0.553	8.817
Charlson Comorbidity Index	0.363	0.160	5.171	1	0.023	1.438	1.051	1.967
GOLD stage (reference)			6.574	2	0.037			
GOLD stage 4	2.257	0.992	5.179	1	0.023	9.557	1.368	66.773
GOLD stage unknown	2.827	1.137	6.178	1	0.013	16.890	1.818	156.909
Infectious AECOPD	1.997	0.657	9.240	1	0.002	7.365	2.033	26.687
SOFA score	0.339	0.109	9.584	1	0.002	1.403	1.132	1.739
Constant	-8.202	1.978	17.188	1	<0.001	0.000		
Abbreviations: AECOPD = Acute Exacerbation of COPD; C.I. = confidence interval; df = degrees of freedom; S.E. = standard error; SOFA = Sequential Organ Failure Assessment								

Table S3 Multivariable binary regression model for neutrophils.

**All AECOPD, Method: Enter, Nagelkerke R^2: 0.598, Hosmer–Lemeshow test p = 0.6, Overall prediction: 84.1%**								
							95% C.I.for EXP(B)	
	B	S.E.	Wald	df	*p* value	Exp(B)	Lower	Upper
Neutrophils >13.45	1.432	0.691	4.294	1	0.038	4.188	1.081	16.229
Gender	0.961	0.732	1.723	1	0.189	2.614	0.622	10.979
Charlson Comorbidity Index	0.330	0.158	4.348	1	0.037	1.391	1.020	1.896
GOLD stage (reference)			6.787	2	0.034			
GOLD stage 4	2.399	1.019	5.536	1	0.019	11.008	1.493	81.183
GOLD stage unknown	2.870	1.146	6.270	1	0.012	17.636	1.866	166.720
Infectious AECOPD	1.904	0.664	8.216	1	0.004	6.713	1.826	24.677
SOFA score	0.338	0.112	9.153	1	0.002	1.402	1.126	1.745
Constant	-8.238	1.994	17.068	1	<0.001	0.000		
Abbreviations: AECOPD = Acute Exacerbation of COPD; C.I. = confidence interval; df = degrees of freedom; S.E. = standard error; SOFA = Sequential Organ Failure Assessment								

Table S4 Multivariable binary regression model for dNLR.

**All AECOPD, Method: Enter, Nagelkerke R square: 0.577, Hosmer–Lemeshow test p = 0.17, Overall prediction: 85.2%**								
							95% C.I.for EXP(B)	
	B	S.E.	Wald	df	*p* value	Exp(B)	Lower	Upper
dNLR >10.53	1.309	0.940	1.940	1	0.164	3.703	0.587	23.358
Gender	0.391	0.698	0.314	1	0.575	1.479	0.376	5.809
Charlson Comorbidity Index	0.278	0.158	3.111	1	0.078	1.321	0.970	1.800
GOLD stage (reference)			6.902	2	0.032			
GOLD stage 4	2.715	1.132	5.750	1	0.016	15.098	1.642	138.823
GOLD stage unknown	3.147	1.228	6.565	1	0.010	23.268	2.095	258.391
Infectious AECOPD	1.927	0.676	8.135	1	0.004	6.869	1.827	25.819
SOFA score	0.345	0.110	9.771	1	0.002	1.412	1.137	1.752
Constant	-7.737	1.928	16.109	1	<0.001	0.000		
Abbreviations: AECOPD = Acute Exacerbation of COPD; C.I. = confidence interval; df = degrees of freedom; dNLR = derived Neutrophil to Lymphocyte Ratio; S.E. = standard error; SOFA = Sequential Organ Failure Assessment								

Table S5 Multivariable binary regression model for NLR.

**All AECOPD, Method: Enter, Nagelkerke R square: 0.575, Hosmer–Lemeshow test p = 0.82, Overall prediction: 79.5%**								
							95% C.I.for EXP(B)	
	B	S.E.	Wald	df	*p* value	Exp(B)	Lower	Upper
NLR >10.12	0.990	0.729	1.846	1	0.174	2.691	0.645	11.224
Gender	0.665	0.689	0.933	1	0.334	1.945	0.504	7.504
Charlson Comorbidity Index	0.315	0.154	4.211	1	0.040	1.371	1.014	1.852
GOLD stage (reference)			6.318	2	0.042			
GOLD stage 4	2.055	0.967	4.517	1	0.034	7.809	1.174	51.965
GOLD stage unknown	2.759	1.115	6.126	1	0.013	15.785	1.776	140.323
Infectious AECOPD	1.834	0.689	7.081	1	0.008	6.261	1.621	24.176
SOFA score	0.337	0.108	9.816	1	0.002	1.401	1.135	1.730
Constant	-7.891	1.905	17.160	1	<0.001	0.000		
Abbreviations: AECOPD = Acute Exacerbation of COPD; C.I. = confidence interval; df = degrees of freedom; NLR = Neutrophil to Lymphocyte Ratio; S.E. = standard error; SOFA = Sequential Organ Failure Assessment								

Table S6 Multivariable binary regression model for NPR.

All AECOPD, Method: Enter, Nagelkerke R^2: 0.608, Hosmer–Lemeshow test p = 0.84, Overall prediction: 79.5%								
							95% C.I.for EXP(B)	
	B	S.E.	Wald	df	*p* value	Exp(B)	Lower	Upper
NPR >0.061	1.630	0.704	5.356	1	0.021	5.106	1.284	20.309
Gender	0.759	0.717	1.119	1	0.290	2.136	0.524	8.709
Charlson Comorbidity Index	0.426	0.173	6.107	1	0.013	1.532	1.092	2.148
GOLD stage (reference)			5.968	2	0.051			
GOLD stage 4	2.440	1.084	5.063	1	0.024	11.473	1.370	96.087
GOLD stage unknown	2.860	1.218	5.511	1	0.019	17.464	1.603	190.212
Infectious AECOPD	1.978	0.664	8.862	1	0.003	7.227	1.965	26.578
SOFA score	0.325	0.114	8.070	1	0.004	1.384	1.106	1.732
Constant	-8.584	2.142	16.059	1	<0.001	0.000		
Abbreviations: AECOPD = Acute Exacerbation of COPD; C.I. = confidence interval; df = degrees of freedom; NPR = Neutrophil to Platelet Ratio; S.E. = standard error; SOFA = Sequential Organ Failure Assessment								

Table S7 Multivariable binary regression model for SII.

All AECOPD, Method: Enter, Nagelkerke R^2: 0.568, Hosmer–Lemeshow test p = 0.26, Overall prediction: 83%								
							95% C.I.for EXP(B)	
	B	S.E.	Wald	df	*p* value	Exp(B)	Lower	Upper
SII >3459	0.678	0.652	1.082	1	0.298	1.970	0.549	7.068
Gender	0.603	0.687	0.771	1	0.380	1.827	0.476	7.019
Charlson Comorbidity Index	0.327	0.154	4.521	1	0.033	1.387	1.026	1.875
GOLD stage (reference)			6.668	2	0.036			
GOLD stage 4	2.138	0.950	5.070	1	0.024	8.487	1.319	54.593
GOLD stage unknown	2.764	1.101	6.303	1	0.012	15.865	1.833	137.284
Infectious AECOPD	1.937	0.690	7.875	1	0.005	6.936	1.793	26.828
SOFA score	0.351	0.108	10.571	1	0.001	1.420	1.149	1.754
Constant	-7.741	1.862	17.290	1	<0.001	0.000		
Abbreviations: AECOPD = Acute Exacerbation of COPD; C.I. = confidence interval; df = degrees of freedom; S.E. = standard error; SII = Systemic Inflammation Index; SOFA = Sequential Organ Failure Assessment								

Table S8 Multivariable binary regression model for MLR.

All AECOPD, Method: Enter, Nagelkerke R^2: 0.587, Hosmer–Lemeshow test p = 0.32, Overall prediction: 77.3%								
							95% C.I.for EXP(B)	
	B	S.E.	Wald	df	*p* value	Exp(B)	Lower	Upper
MLR >1.054	1.160	0.647	3.220	1	0.073	3.191	0.898	11.332
Gender	0.476	0.705	.455	1	0.500	1.609	0.404	6.410
Charlson Comorbidity Index	0.376	0.164	5.256	1	0.022	1.457	1.056	2.010
GOLD stage (reference)			6.810	2	0.033			
GOLD stage 4	2.357	0.993	5.630	1	0.018	10.559	1.507	73.984
GOLD stage unknown	2.762	1.109	6.204	1	0.013	15.835	1.802	139.187
Infectious AECOPD	2.190	0.667	10.794	1	0.001	8.938	2.420	33.016
SOFA score	0.321	0.106	9.241	1	0.002	1.378	1.121	1.695
Constant	-8.076	1.955	17.057	1	<0.001	0.000		

Abbreviations: AECOPD = Acute Exacerbation of COPD; C.I. = confidence interval; df = degrees of freedom; MLR = Monocyte to Lymphocyte Ratio; S.E. = standard error; SOFA = Sequential Organ Failure Assessment

Table S9 Multivariable binary regression model for SIRI.

All AECOPD, Method: Enter, Nagelkerke R^2: 0.622, Hosmer–Lemeshow test p = 0.06, Overall prediction: 84.1%								
							95% C.I. for EXP(B)	
	B	S.E.	Wald	df	*p* value	Exp(B)	Lower	Upper
SIRI >15.27	1.859	0.717	6.726	1	0.009	6.419	1.575	26.160
Gender	0.768	0.724	1.124	1	0.289	2.156	0.521	8.915
Charlson Comorbidity Index	0.448	0.180	6.198	1	0.013	1.565	1.100	2.227
GOLD stage (reference)			6.375	2	0.041			
GOLD stage 4	2.398	1.033	5.387	1	0.020	11.005	1.452	83.393
GOLD stage unknown	2.843	1.184	5.764	1	0.016	17.168	1.686	174.855
Infectious AECOPD	1.996	0.694	8.276	1	0.004	7.360	1.889	28.674
SOFA score	0.335	0.112	8.883	1	0.003	1.397	1.121	1.741
Constant	-8.813	2.196	16.109	1	<0.001	0.000		

Abbreviations: AECOPD = Acute Exacerbation of COPD; C.I. = confidence interval; df = degrees of freedom; S.E. = standard error; SIRI = Systemic Inflammation Response Index; SOFA = Sequential Organ Failure Assessment

Table S10 Multivariable binary regression model for haematocrit.

**All AECOPD, Method: Enter, Nagelkerke R^2: 0.565, Hosmer–Lemeshow test p = 0.7, Overall prediction: 83%**								
							95% C.I.for EXP(B)	
	B	S.E.	Wald	df	*p* value	Exp(B)	Lower	Upper
Haematocrit <43.5	0.649	0.728	0.794	1	0.373	1.914	0.459	7.978
Gender	0.816	0.704	1.345	1	0.246	2.262	0.569	8.989
Charlson Comorbidity Index	0.253	0.169	2.231	1	0.135	1.288	0.924	1.795
GOLD stage (reference)			6.036	2	0.049			
GOLD stage 4	2.091	0.979	4.559	1	0.033	8.089	1.187	55.128
GOLD stage unknown	2.686	1.116	5.789	1	0.016	14.675	1.645	130.876
Infectious AECOPD	2.070	0.656	9.969	1	0.002	7.928	2.193	28.664
SOFA score	0.349	0.106	10.894	1	<0.001	1.417	1.152	1.743
Constant	-7.659	1.878	16.633	1	<0.001	0.000		
Abbreviations: AECOPD = Acute Exacerbation of COPD; C.I. = confidence interval; df = degrees of freedom; S.E. = standard error; SOFA = Sequential Organ Failure Assessment								

Table S11 Multivariable binary regression model for MCHC.

**All AECOPD, Method: Enter, Nagelkerke R^2: 0.612, Hosmer–Lemeshow test p = 0.62, Overall prediction: 85.2%**								
							95% C.I. for EXP(B)	
	B	S.E.	Wald	df	*p* value	Exp(B)	Lower	Upper
MCHC >31.5	1.630	0.677	5.789	1	0.016	5.102	1.353	19.241
Gender	0.647	0.746	0.754	1	0.385	1.911	0.443	8.241
Charlson Comorbidity Index	0.326	0.160	4.155	1	0.042	1.386	1.013	1.897
GOLD stage (reference)			5.514	2	0.063			
GOLD stage 4	2.379	1.070	4.945	1	0.026	10.791	1.326	87.822
GOLD stage unknown	2.607	1.199	4.730	1	0.030	13.554	1.294	141.998
Infectious AECOPD	2.080	0.664	9.803	1	0.002	8.003	2.177	29.425
SOFA score	0.386	0.120	10.362	1	0.001	1.472	1.163	1.862
Constant	-8.434	2.119	15.848	1	<0.001	0.000		
Abbreviations: AECOPD = Acute Exacerbation of COPD; C.I. = confidence interval; df = degrees of freedom; MCHC = Mean Corpuscular Haemoglobin Concentration; S.E. = standard error; SOFA = Sequential Organ Failure Assessment								

2. Multivariable binary regressions for the studied hematological parameters in non-infectious AECOPD patients

Table S12 Multivariable binary regression model for SIRI.

Non-infectious AECOPD, Method: Enter, Nagelkerke R^2: 0.633, Hosmer–Lemeshow test p = 0.73, Overall prediction: 87.5%								
							95% C.I.for EXP(B)	
	B	S.E.	Wald	df	*p* value	Exp(B)	Lower	Upper
SIRI > 10.96	4.146	1.651	6.307	1	0.012	63.172	2.485	1605.872
Glasgow Coma Scale	-0.371	0.182	4.129	1	0.042	0.690	0.483	0.987
Gender	1.067	1.456	0.537	1	0.464	2.908	0.167	50.492
Charlson Comorbidity Index	0.392	0.366	1.144	1	0.285	1.479	0.722	3.031
Constant	-2.305	2.575	0.801	1	0.371	0.100		
Abbreviations: AECOPD = Acute Exacerbation of COPD; C.I. = confidence interval; df = degrees of freedom; S.E. = standard error; SIRI = Systemic Inflammation Response Index								

Table S13 Multivariable binary regression model for SII.

Non-infectious AECOPD, Method: Enter, Nagelkerke R^2: 0.528, Hosmer–Lemeshow test p = 0.91, Overall prediction: 85%								
							95% C.I.for EXP(B)	
	B	S.E.	Wald	df	*p* value	Exp(B)	Lower	Upper
SII >4588	3.205	1.318	5.913	1	0.015	24.646	1.862	326.185
Glasgow Coma Scale	-0.211	0.119	3.159	1	0.075	0.810	0.641	1.022
Gender	2.023	1.511	1.791	1	0.181	7.557	0.391	146.124
Charlson Comorbidity Index	0.478	0.360	1.765	1	0.184	1.613	0.797	3.264
Constant	-3.876	2.731	2.015	1	0.156	0.021		
Abbreviations: AECOPD = Acute Exacerbation of COPD; C.I. = confidence interval; df = degrees of freedom; S.E. = standard error; SII = Systemic Inflammation Index								

Table S14 Multivariable binary regression model for monocytes.

Non-infectious AECOPD, Method: Enter, Nagelkerke R^2: 0.465, Hosmer–Lemeshow test p = 0.9, Overall prediction: 87.5%								
							95% C.I. for EXP(B)	
	B	S.E.	Wald	df	*p* value	Exp(B)	Lower	Upper
Monocytes >1	2.268	1.011	5.030	1	0.025	9.663	1.331	70.148
Glasgow Coma Scale	-0.190	0.117	2.649	1	0.104	0.827	0.657	1.040
Gender	1.562	1.338	1.362	1	0.243	4.769	0.346	65.728
Charlson Comorbidity Index	0.409	0.312	1.721	1	0.190	1.506	0.817	2.775
Constant	-3.435	2.284	2.261	1	0.133	0.032		

Abbreviations: AECOPD = Acute Exacerbation of COPD; C.I. = confidence interval; df = degrees of freedom; S.E. = standard error

3. **Multivariable binary regressions for the studied hematological parameters in infectious AECOPD patients**

Table S15 Multivariable binary regression model for platelets.

**Infectious AECOPD, Method: Enter, Nagelkerke R^2: 0.496, Hosmer–Lemeshow test p = 0.07, Overall prediction: 77.1%**								
							95% C.I. for EXP(B)	
	B	S.E.	Wald	df	*p* value	Exp(B)	Lower	Upper
Platelets <209	1.284	0.931	1.902	1	0.168	3.613	0.582	22.414
Gender	-0.322	0.892	0.131	1	0.718	0.724	0.126	4.164
Age	-0.002	0.041	0.003	1	0.956	.998	0.920	1.081
LVHF	1.822	1.023	3.173	1	0.075	6.183	0.833	45.904
Modified SOFA	0.526	0.184	8.184	1	0.004	1.692	1.180	2.425
Constant	-3.187	2.823	1.275	1	0.259	0.041		

Abbreviations: AECOPD = Acute Exacerbation of COPD; C.I. = confidence interval; df = degrees of freedom; LVHF = left ventricle heart failure; S.E. = standard error; SOFA = Sequential Organ Failure Assessment
Modified SOFA refers to the exclusion of points given if the subject presented thrombocytopaenia. This was done in order to reduce the multicollinearity between variables 'Platelets' and 'SOFA'

Table S16 Multivariable binary regression model for MCHC.

**Infectious AECOPD, Method: Enter, Nagelkerke R^2: 0.625, Hosmer–Lemeshow test p = 0.68, Overall prediction: 83.3%**								
							95% C.I. for EXP(B)	
	B	S.E.	Wald	df	*p* value	Exp(B)	Lower	Upper
MCHC >31.5	2.787	1.111	6.294	1	0.012	16.224	1.840	143.087
Gender	0.046	0.977	0.002	1	0.962	1.048	0.154	7.113
Age	-0.049	0.041	1.429	1	0.232	0.952	0.879	1.032
LVHF	1.612	1.165	1.914	1	0.167	5.013	0.511	49.193
SOFA	0.548	0.205	7.164	1	0.007	1.730	1.158	2.583
Constant	-0.932	2.614	0.127	1	0.721	0.394		

Abbreviations: AECOPD = Acute Exacerbation of COPD; C.I. = confidence interval; df = degrees of freedom; LVHF = left ventricle heart failure; MCHC = Mean Corpuscular Haemoglobin Concentration; S.E. = standard error; SOFA = Sequential Organ Failure Assessment

Table S17 Multivariable binary regression model for NPR.

Infectious AECOPD, Method: Enter, Nagelkerke R^2: 0.564, Hosmer–Lemeshow test p = 0.84, Overall prediction: 79.2%								
							95% C.I.for EXP(B)	
	B	S.E.	Wald	df	*p* value	Exp(B)	Lower	Upper
NPR >0.0657	1.925	0.959	4.033	1	0.045	6.855	1.047	44.871
Gender	-0.412	0.896	0.211	1	0.646	0.663	0.114	3.838
Age	-0.039	0.040	0.931	1	0.335	0.962	0.890	1.041
LVHF	2.308	1.105	4.362	1	0.037	10.050	1.153	87.623
SOFA	0.570	0.208	7.489	1	0.006	1.768	1.176	2.659
Constant	-1.289	2.489	0.268	1	0.604	0.275		
Abbreviations: AECOPD = Acute Exacerbation of COPD; C.I. = confidence interval; df = degrees of freedom; LVHF = left ventricle heart failure; NPR = Neutrophil to Platelet Ratio; S.E. = standard error; SOFA = Sequential Organ Failure Assessment								

4. **Multivariable binary regressions for the studied hematological parameters in bacterial AECOPD patients**

Table S18 Multivariable binary regression model for leukocytes.

Bacterial AECOPD, Method: Enter, Nagelkerke R^2: 0.599, Hosmer–Lemeshow test p = 0.33, Overall prediction: 77.5%								
							95% C.I.for EXP(B)	
	B	S.E.	Wald	df	*p* value	Exp(B)	Lower	Upper
Leukocytes >11.08	2.479	1.170	4.484	1	0.034	11.925	1.203	118.247
Gender	0.968	1.053	0.844	1	0.358	2.631	0.334	20.725
Charlson Comorbidity Index	0.183	0.186	0.969	1	0.325	1.201	.834	1.730
SOFA score	0.596	0.207	8.337	1	0.004	1.816	1.211	2.722
Constant	-6.555	2.434	7.253	1	0.007	0.001		
Abbreviations: AECOPD = Acute Exacerbation of COPD; C.I. = confidence interval; df = degrees of freedom; S.E. = standard error; SOFA = Sequential Organ Failure Assessment								

Table S19 Multivariable binary regression model for neutrophils.

Bacterial AECOPD, Method: Enter, Nagelkerke R^2: 0.583, Hosmer–Lemeshow test p = 0.18, Overall prediction: 77.5%								
							95% C.I. for EXP(B)	
	B	S.E.	Wald	df	*p* value	Exp(B)	Lower	Upper
Neutrophils >13.58	2.151	1.057	4.140	1	0.042	8.595	1.082	68.255
Gender	1.390	1.143	1.478	1	0.224	4.014	0.427	37.737
Charlson Comorbidity Index	0.108	0.179	0.363	1	0.547	1.114	0.784	1.582
SOFA score	0.579	0.203	8.111	1	0.004	1.784	1.198	2.657
Constant	-5.830	2.206	6.985	1	0.008	0.003		
Abbreviations: AECOPD = Acute Exacerbation of COPD; C.I. = confidence interval; df = degrees of freedom; S.E. = standard error; SOFA = Sequential Organ Failure Assessment								

Table S20 Multivariable binary regression model for NPR.

Bacterial AECOPD, Method: Enter, Nagelkerke R^2: 0.594, Hosmer–Lemeshow test p = 0.82, Overall prediction: 75%								
							95% C.I. for EXP(B)	
	B	S.E.	Wald	df	*p* value	Exp(B)	Lower	Upper
NPR >0.0657	2.182	1.028	4.508	1	0.034	8.862	1.183	66.408
Gender	0.800	1.058	0.572	1	0.450	2.225	0.280	17.701
Charlson Comorbidity Index	0.203	0.186	1.192	1	0.275	1.224	0.851	1.761
SOFA score	0.493	0.198	6.192	1	0.013	1.637	1.110	2.413
Constant	-4.958	1.940	6.533	1	0.011	0.007		
Abbreviations: AECOPD = Acute Exacerbation of COPD; C.I. = confidence interval; df = degrees of freedom; NPR = Neutrophil to Platelet Ratio; S.E. = standard error; SOFA = Sequential Organ Failure Assessment								

Table S21 Multivariable binary regression model for MCHC.

Bacterial AECOPD, Method: Enter, Nagelkerke R^2: 0.605, Hosmer–Lemeshow test p = 0.73, Overall prediction: 85%								
							95% C.I. for EXP(B)	
	B	S.E.	Wald	df	*p* value	Exp(B)	Lower	Upper
MCHC >31.55	2.409	1.129	4.550	1	0.033	11.121	1.216	101.708
Gender	0.366	1.039	0.124	1	0.724	1.443	0.188	11.060
Charlson Comorbidity Index	0.046	0.210	0.048	1	0.826	1.047	0.694	1.580
SOFA score	0.580	0.207	7.890	1	0.005	1.786	1.192	2.678
Constant	-4.466	1.908	5.479	1	0.019	0.011		
Abbreviations: AECOPD = Acute Exacerbation of COPD; C.I. = confidence interval; df = degrees of freedom; MCHC = Mean Corpuscular Haemoglobin Concentration; S.E. = standard error; SOFA = Sequential Organ Failure Assessment								
